# Exploring the ethics of tuberculosis human challenge models

**DOI:** 10.1136/jme-2023-109234

**Published:** 2023-12-30

**Authors:** Abie Rohrig, Josh Morrison, Gavriel Kleinwaks, Jonathan Pugh, Helen McShane, Julian Savulescu

**Affiliations:** 1 Columbia University, New York, New York, USA; 2 1Day Sooner, Baltimore, Maryland, USA; 3 Oxford Uehiro Centre for Practical Ethics, University of Oxford, Oxford, UK; 4 Jenner Institute, University of Oxford Nuffield Department of Medicine, Oxford, UK; 5 Centre for Biomedical Ethics, Yong Loo Lin School of Medicine, National University of Singapore, Singapore; 6 Biomedical Research Group, Murdoch Childrens Research Institute, Melbourne, Victoria, Australia

## Abstract

We extend recent conversation about the ethics of human challenge trials to tuberculosis (TB). TB challenge studies could accelerate vaccine development, but ethical concerns regarding risks to trial participants and third parties have been a limiting factor. We analyse the expected social value and risks of different challenge models, concluding that if a TB challenge trial has between a 10% and a 50% chance of leading to the authorisation and near-universal delivery of a more effective vaccine 3–5 years earlier, then the trial would save between 26 400 and 1 100 000 lives over the next 10 years. We also identify five important ethical considerations that differentiate TB from recent human challenge trials: an exceptionally high disease burden with no highly effective vaccine; heightened third party risk following the trial, and, partly for that reason, uniquely stringent biosafety requirements for the trial; risks associated with best available TB treatments; and difficulties with TB disease detection. We argue that there is good reason to consider conducting challenge trials with attenuated strains like Bacillus Calmette-Guérin or attenuated *Mycobacterium tuberculosis*.

## Background

### Human challenge trials

Human challenge trials (also referred to as ‘human infection studies’), studies in which volunteers are deliberately exposed to a pathogen, have contributed vital scientific knowledge to advance vaccine development in recent decades. Challenge models have been used for a wide range of diseases, including malaria, influenza and most recently COVID-19. Studies involving deliberate infection are particularly useful when field studies would be lengthy and expensive. They can help reduce uncertainty in the early stages of vaccine development by allowing for the optimal allocation of resources toward the most promising vaccine candidates, reducing the costs of clinical development and encouraging investment in larger-scale trials.^1^

Supplementary questions regarding vaccination, such as optimal method of administration and vaccine dosage, can also be investigated through challenge studies. More broadly, challenge studies can advance scientific understanding about pathogens by revealing precise data on disease pathogenesis, correlates of protection (or in other words, the biomarkers of immunity), viral kinetics and shedding.^2^

In this paper, we draw on recent ethical frameworks to analyse the ethics of tuberculosis (TB) human challenge trials.

### The state of TB vaccine development and TB human challenge trials

The only available TB vaccine, Bacillus Calmette-Guérin (BCG), was developed and licensed nearly 100 years ago. Despite nearly universal BCG coverage in TB-endemic regions, TB caused 1.6 million deaths in 2021, more than any other pathogen.^3^ This is in part because BCG is just 19% effective at preventing infection in children, and 58% effective at preventing disease.^4^ Effectiveness tends to wane almost entirely in adolescence and adulthood, leading to significant death rates among adults ages 50 and older, although there are some areas in which BCG confers durable protection.^5^

Several new TB vaccine approaches that may have advantages over a single infant BCG vaccination have recently shown promise in clinical trials. First, a Phase 2b trial showed that M72/AS01E, a combination of two *Mycobacterium tuberculosis* (*M. tb*) protein fragments, protected adults with latent *M.tb* infection (LTBI) from disease with an efficacy rate of 50% compared with unvaccinated adults with LTBI.^6^ On 28 June 2023, the Gates Foundation and the Wellcome Trust announced US$550 million in support of a Phase 3 trial to test M72/AS01E. Second, a Phase 2 trial showed that a BCG booster dose was 45% efficacious at reducing sustained *M.tb* infection.^7^ However, it remains to be seen whether BCG revaccination protects against disease, and one study has shown that it did not.^8^ While no other TB vaccine candidates have yet shown efficacy in clinical trials, several other vaccine candidates are in the pipeline, including MTBVAC, which is based on a live-attenuated strain of *M.tb* and could therefore possibly be of use as a challenge agent.^9^

These vaccine candidates may significantly reduce TB disease burden, and new vaccine candidates that have not yet entered trials may prove even more effective.

However, there are currently several barriers to TB vaccine research. Animal challenge models do not adequately represent the complexity of *M.tb* infection and TB disease in humans. The availability of *M.tb* challenge models in mice, guinea pigs, cattle, rabbits and non-human primates have elucidated certain pathways in pathogen–host interaction, but none capture all aspects of human TB.^10^

Additionally, Phase 3 trials to gauge TB vaccine efficacy have several limitations. They take several years on average, allowing for millions of TB deaths in the meantime. Large Phase 3 trials are also expensive: a recent rotavirus Phase 3 trial cost over US$100 million to conduct,^10^ and the upcoming M72/AS01E trial is expected to cost US$550 million. Since TB primarily affects the global poor, a lack of funding has been a consistent obstacle for TB vaccine research. It has taken around 20 years for M72/AS01E to reach a Phase 3 trial. In an ideal world, we would have ample funding for TB vaccine research, but given the limited available funding, we must consider alternative trial methods that allow us to make the most use of the few Phase 3 trials that are likely to be funded.

Human challenge trials can address the pitfalls of Phase 3 trials by allowing for quick reads on a candidate vaccine’s efficacy in preventing infection; in turn, this can provide crucial information for decisions about which candidate vaccines to prioritise for larger-scale trials. A single TB vaccine candidate could be tested directly against the current BCG vaccine, or, given that BCG-induced immunity wanes by adulthood and a human challenge trial would recruit young adults, a candidate could be tested directly against a placebo. Multiple novel TB vaccines could be added in a multiarm study. Reducing uncertainty in the early stages of vaccine development can be of enormous utility in convincing funders and stakeholders that Phase 3 trials are worth the time and investment.^1^

In particular, human challenge trials are a useful way of selecting which vaccine candidates should progress to field efficacy studies. Investors who may not otherwise fund larger efficacy studies may be convinced to do so if a vaccine candidate performed well in challenge studies, shaving years off of vaccine development. For instance, challenge studies have been used to successfully select malaria vaccines.^11^

In the context of TB, selection of leading vaccine candidates and additional funding for Phase 3 trials are especially important. Over a dozen TB vaccine candidates are in the pipeline, none of which have begun a Phase 3 trial.^12^ It is possible that eventually, efficacy data from challenge studies, in combination with efficacy data from Phase 2b trials and safety data from a larger study, may be sufficient for vaccine authorisation, obviating the need for a lengthy Phase 3 trial.

Unlike other infectious diseases such as influenza, there is no identified immune correlate of protection for TB, making authorisation by surrogate endpoint impossible and limiting early estimates on vaccine efficacy. While correlates of protection can be identified in Phase 3 trials of an effective TB vaccine by collecting blood samples post-vaccination, the identification and subsequent use of correlates of protection can be aided by human challenge trials in two ways. First, in the absence of Phase 3 trials, human challenge trials can be used to identify correlates of protection in vaccine efficacy studies more quickly and on a smaller scale by collecting blood samples post-vaccination and pre-challenge. Given the length and expense of Phase 3 trials, this avenue may practically be very important. Second, if a field study is already occurring, a human challenge model could be validated against the field study outcome, showing that the human challenge model reliably reflects field outcomes and possibly allowing for the testing of future vaccines without Phase 3 studies.

We note that a multiarm human challenge study would require significantly more costs and volunteers than a single-arm study, especially because the participants will need to be housed in a biosafe facility. However, given that challenge trials typically involve dozens of volunteers and field studies typically involve thousands of volunteers, we estimate that a multiarm challenge trial would still be significantly cheaper than a multiarm field study. Additionally, we note that one limitation of human challenge trials compared with field studies is that the former can only ethically use infection as a clinical endpoint rather than disease. While preventing infection would be a massive step toward reducing TB disease burden, it would not be able to help those who already have latent TB. However, although disease cannot be an endpoint in a challenge study, a biological signal of efficacy against infection in a controlled human infection model may increase confidence in moving a candidate forward in a prevention-of-disease efficacy trial.

## The social value and risks of a TB human challenge model

In light of the above considerations, we can now begin to assess whether TB human challenge models might be ethically warranted. We begin our analysis by considering whether such a trial could have a favourable ratio of societal benefits to risks facing trial participants and third parties. To be clear at the outset, we consider a favourable benefit to risk ratio to be a necessary but insufficient prerequisite for a trial to be ethically permissible.^13^

### Social value of a TB human challenge model

Recent frameworks from Shah *et al* and the WHO Working Group for Guidance on Human Challenge Studies in COVID-19 emphasise that in determining the ethical status of a human challenge trial, a key first step is to gauge its social value.^14 15^

Rid and Roestenberg provide a framework to determine the expected social value of human challenge trials by considering both (1) the magnitude of the disease health burden that the trial would address, and (2) the probability that a trial contributes to the mitigation of this disease burden.^16^ In the rest of this section, we describe the global TB disease burden and offer a range of estimates for how many lives a TB challenge model might save.

#### Magnitude of TB disease burden

In 2019, TB caused an estimated 10 million illnesses and 1.4 million deaths, making it the world’s deadliest pathogen and the leading cause of death for people with HIV. TB overwhelmingly affects the global poor, contributing to cycles of poverty in which TB illness reduces economic mobility. Crowded conditions and impaired immune function associated with poverty lead to greater disease spread. Lengthy treatment regimens contribute to this cycle, with a meta-analysis of patients with TB in Africa finding that direct and indirect medical costs associated with TB are substantial and often ‘catastrophic’ for those in the income-poorest 20% of the population.^17^

Several public health interventions have contributed to a 14% decrease in TB deaths from 2015 to 2019. These include increased diagnosis, drug susceptibility testing, preventative treatment for high-risk populations (including those who are HIV-infected) and the mitigation of environmental determinants of TB (such as poverty).^18^ Despite these efforts, few countries have met the 2020 milestones set out by the WHO’s End TB Strategy, with the poorest countries falling behind the most.

Given the slow pace of TB control efforts, it is clear that the authorisation and large-scale distribution of a more effective TB vaccine are important for significant reductions in TB incidence and death in the coming decade.

#### Probability that a TB challenge trial contributes to TB mitigation

We now turn to estimating the expected social value of a TB human challenge trial by discerning the likelihood that such a study would contribute to the speedier authorisation of more effective TB vaccines.

Rid and Roestenberg outline several crucial considerations for making this judgement, while noting the complexity and uncertainty of determinations of social value made before a trial begins. These considerations include the novelty and innovation of research questions, feasibility and rigour of research conduct and influence on future research with the potential to lead to health benefits.^16^

Leading TB vaccinologists agree that establishing a TB challenge model would significantly aid in vaccine development.^19^ Many of the benefits of challenge trials identified in the introduction would be applicable to the context of a TB vaccine. These include the speedy testing of promising vaccine candidates for selection and optimal resource reallocation, reducing uncertainty in early-stage vaccine development to encourage investment in larger-scale TB efficacy trials, the identification of an immune correlate of TB protection to enable future vaccine testing via surrogate endpoint, the optimisation of vaccine route of administration and dosage size and the study of TB pathogenesis.

Several TB challenge models are still in development and thus may not be feasible for several years. Given the difficulty of reliably and accurately quantifying BCG in the bronchoalveolar lavage fluid, an alternative mycobacterial challenge approach uses a skin challenge and punch biopsy model, in which BCG is injected into the skin and subsequently quantified accurately from the punch biopsy. This model is easier to deliver, but does not mimic the natural route of infection. Developing a pulmonary model may require future advances in diagnostic development and methods for quantifying BCG and other mycobacteria in the lung. However, given the slow pace of traditional TB vaccine development and the consistently high disease burden of TB, challenge models that are established in the near future will likely still have significant utility.

In table 1, we offer low, middle and high-end estimates of the lives saved in expectation through a TB challenge model, assuming an average rate of total TB deaths over the next decade of approximately 1.1 million per year and an 80% coverage rate of TB vaccines^†^. For instance, in our middle-end estimate, we say that if a given TB challenge model is 25% more likely to speed the authorisation of a 30% more efficacious TB vaccine by 4 years, then the challenge model will save 264 000 lives in expectation. We explain and justify the assumptions found in table 1 in online supplemental appendix A.

Table 1 has several limitations. Above all, the ever-changing nature of disease burden means that our assumptions may not be accurate at the time when researchers are deciding whether to conduct a specific TB challenge trial. Since there is no rigorous mathematical modelling of the public health effects of speeding the authorisation of the two most promising next-generation TB vaccines, namely M72/AS01E and BCG revaccination, we employed simple probabilities for estimates for vaccine efficacy and the speed of vaccine rollout. Moreover, our low, middle and high-end estimates correlate specific probabilities that a TB challenge model speeds vaccine research with specific probabilities that such a vaccine is more or less effective, but there is no a priori reason why these figures should be correlated.

Despite its limitations, we think table 1 illustrates the plausible upper and lower bounds of the range of numbers of lives that might be saved by. In fact, the table is likely conservative in its estimation of the reduction of disease burden in these trials for three reasons. First, it does not account for the full range of TB disease burden, such as morbidity, cost of treatment and macroeconomic effects. Second, it focuses solely on the short-term value of challenge models in speeding vaccine development, not accounting for the long-run value of possibly discerning correlates of TB immune protection, which could aid TB vaccine and treatment development for decades to come. Third, it does not account for the compounding effect of TB vaccination in reducing TB deaths by curbing disease transmission. For a fuller picture of the benefits of accelerating TB vaccination, see Clark *et al*.^20^

The expected social value of a TB human challenge trial is largely dependent on the specific type of trial. Table 2 offers a breakdown of some use cases. In the next section, we discuss the risks of conducting different types of TB human challenges.

### Risks of a TB human challenge model

Recent WHO ethical guidance on TB human challenge studies states that ‘despite the substantial benefits that TB [human challenge studies] could plausibly be associated with, it is currently unclear whether the risks could ever be justifiable’.^15^ In this section, we analyse the risks of TB, first in general, and then in the context of different types of human challenge trials, with a focus on volunteer risks in attenuated pulmonary *M.tb* challenge models.

#### Risks of TB disease

Unlike recently-conducted COVID-19 human challenge trials that dealt with an emerging disease, we have decades of data about the rates of mortality and morbidity for a given case of TB. On exposure to the pathogen, patients will most likely clear the infection before it becomes latent. There is a small chance of the infection becoming latent, though, which is a symptom-free condition that, without treatment, will turn active 5–10% of the time.^21^ Preventative treatments for those with latent TB, such as anti-TB chemotherapy, reduce the likelihood that latent TB becomes active by 93% assuming full patient compliance with therapy.^22^

**Figure 1:**
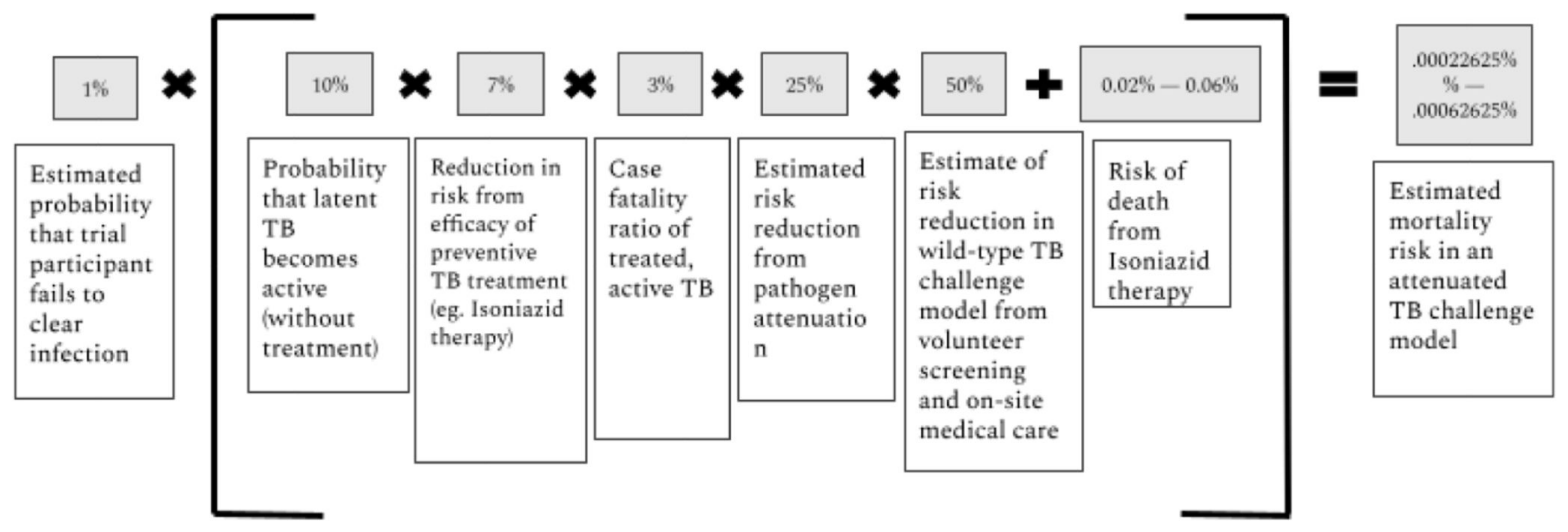
Estimate mortality risk in an attenuated TB challenge model. TB, tuberculosis.

While active TB is curable 90% of the time with an antibiotic cocktail consisting of isoniazid, rifampicin, pyrazinamide and ethambutol,^23^ active TB still leads to death 3% of the time for HIV-negative patients.^24^ Moreover, TB antibiotics carry their own risks: Isoniazid, a primary component of TB treatment, can cause hepatotoxicity which results in death between 0.02% and 0.06% of the time.^25^ In figure 1, we aim to estimate the mortality risks for an individual in an attenuated *M.tb* challenge trial. We begin with the probability that a participant does not clear the infection, as the pathogen would be designed such that it is very unlikely to cause persistent infection. We multiply this by the probability of the risk of infection leading to disease, given treatment. We multiply that with the base rate of mortality caused by active TB, and multiply that by risk reduction variables given the attenuated pathogen, volunteer screening and excellent on-site care, the latter of which was standard in recent UK COVID-19 challenge trials. We then estimate the mortality risk from the treatment, which includes isoniazid therapy. We are left with a mortality estimate of between 0.00023% and 0.00063% (see figure 1).

In addition to short-term mortality risk, well-treated TB disease also involves significant long-term negative health effects. In a study of all-cause mortality for people with treated TB, Romanowski *et al* find ‘significantly increased mortality following treatment compared with the general population or matched controls’.^26^ Pulmonary TB is associated with long-term lung complications, such as lung scarring, bronchiectasis and chronic pulmonary disease. TB treatment itself also carries long-term risks: microbiomic perturbation caused by TB therapy is long-lasting, which can cause and exacerbate other diseases.^27^

One-way in which participation in a TB challenge trial would reduce risks for volunteers from endemic regions is by reducing the risk of reinfection. Reinfected TB individuals have a 79% lower risk of active disease than uninfected individuals.^28^ Active TB has an R naught between 0.23 and 4.3.^29^ Each TB case therefore carries a significant risk of community transmission. However, this risk can be minimised through active monitoring of at-risk individuals and subsequent safety procedures, as discussed in the following section.

#### Risks in the context of an attenuated TB human challenge model

In the context of a human challenge study, TB risks to volunteers are likely to be significantly lower than risks to individuals typically infected with TB in endemic regions for three reasons. The first reason is the attenuation of the pathogen itself; the probability of mortality and morbidity would decrease on exposure to a strain of *M.tb* that has a limited period of replication and/or regulated expression of kill switches. Such an attenuated model could significantly reduce the chances of latent TB infection becoming active long after the commencement of the study, when a volunteer may be immunocompromised. While it is difficult to estimate a priori exactly how much an attenuated model might reduce the risks of TB, we assume conservatively in figure 1 that the process of attenuation will likely reduce the risk of death in a TB challenge trial by 75%.

Second, researchers can select volunteers in the lowest risk profile, that is, young adult volunteers with no comorbidities. This is likely to reduce risks considerably, as HIV, old age and infancy all increase the risk of TB disease.^27^ Notably, prescreening for healthy volunteers is limited by the possibility that a volunteer ages significantly and becomes immunocompromised after the trial, when they are still susceptible to the activation of latent TB.

Third, unlike many TB-endemic regions which have poor health infrastructure, *M.tb* challenge trial participants will have access to world-class treatments, as well as excellent and timely medical care in the case of any adverse events. We estimate in figure 1 that volunteer prescreening and safety measures in tandem are likely to reduce mortality following *M.tb* infection in an *M.tb* challenge model by 50%. The total estimated mortality risk following *M.tb* infection in an attenuated *M.tb* challenge trial is between 0.00023% and 0.00063%. There is no net health benefit for volunteers for taking part in this research. Nevertheless, in table 3 we show that these risks are on par with other common altruistic and non-altruistic risks taken both in medical contexts, such as living donation and plastic surgery. Risks also fall well below upper bounds of risk in trials; other commentators have put forth a 1% risk of death or severe illness as a suggested upper bound.

The mortality estimation in figure 1 does not capture the full range of risks in an attenuated human challenge trial. For instance, it is important to consider whether eventually-active TB may cause volunteers to be isolated according to local public health law in order to prevent harm to others. As detailed above, there is only an extremely low risk of infection not being cleared, leading to latent TB and even then a low risk of latent TB developing to active TB. If required, containment would be unlikely to be more than 2 weeks as this is what is usually necessary for someone with smear positive TB. We discuss this more in the section entitled third party risk.

Moreover, even an *M.tb* challenge model with low risks of mortality would involve possible risks of long-term morbidity. This can be reduced by using an attenuated strain of TB, as efforts are underway to create an attenuated *M.tb* strain that has a limited period of replication. We argue in the following section that it is not unusual for altruistic individuals to voluntarily incur comparably significant long-term risk for the common good. The exact risks and benefits of a given TB challenge study will depend on trial design and the pathogen in use. Table 2 explores the use cases, limitations, and risks of different TB challenge trials. In the following section, we explore the ethics of virulent *M.tb* human challenge trials.

### When are the benefits of research sufficient to justify its risks?

Up to this point, we have outlined some of the salient general risks and benefits of a TB challenge trial. Of course, it is impossible to determine a priori whether or a given *M.tb* challenge trial would have a favourable risk-benefit ratio in the absence of a detailed trial protocol.

However, in considering whether such a trial could have a favourable risk-benefit ratio, we need to attend to the deeper philosophical question under what conditions the benefits of a research study justify its risks.^30^ Of course, there are more or less complex answers to this question. A simple libertarian approach would claim that any research risk is justifiable if individuals with decision-making capacity give valid consent to expose themselves to those risks; a simple consequentialist approach would claim that the overall expected benefits of the study must outweigh its risks if those risks are to be justifiable. While these approaches would likely speak in favour of the permissibility of TB challenge trials, they do not enjoy widespread support and they are not reflected in standard approaches to understanding risk-benefit analysis in research ethics.

In a similar vein, there are simple approaches to the question that might speak against the permissibility of TB challenge trials relatively straightforwardly. For instance, if research risks cannot be justified unless the participant herself is expected to net benefit from her participation, then it will naturally be difficult to justify challenge trials (although perhaps not impossible). However, an approach requiring participant net benefit is also controversially paternalistic, as it would rule out the permissibility of a great deal of valuable research that might pose low risks that consenting adults are willing to take, even if they do not stand to net benefit from participation.^31^ Indeed, as Miller and Joffe note, we often do not adopt a similarly paternalistic approach with volunteer subjects in public health emergencies.^32^ The WHO declared TB a public health emergency in 1993, and though deaths have decreased since then, they remain higher than any other infectious disease. We think the misery caused by TB therefore warrants the same urgency as other public health emergencies.

One-way of avoiding the pitfalls of these approaches toward limits to research risks is to combine a broadly consequentialist approach with an acceptable risk threshold. On this approach, risks of research can be justifiable if they are outweighed by the benefits of the study and the risks fall below a stipulated maximum threshold. One example of such a threshold is the minimal risk standard; research is deemed to pose minimal risk if its risks do not exceed those encountered in everyday life. This standard is typically invoked as a sorting threshold to determine whether Institutional Review Boards can exercise discretion about expediting review, waiving consent requirements and enrolling vulnerable participants in studies that may not directly benefit them.

However, for research enrolling only participants with decision-making capacity, it may be appropriate to set an acceptable risk threshold that allows for risk in research that goes beyond the merely minimal. There are plausible reasons, grounded in personal autonomy, to respect the choices of individuals to expose themselves to risks, even if we would not expose individuals to those risks without their volunteering. Indeed, we suggest that one plausible candidate for such a higher threshold, as suggested by London, is the level of risk that society allows individuals to expose themselves to (perhaps for altruistic reasons) such as organ donation.^33^

Naturally, the higher the acceptable risk threshold we invoke, the more likely it is that the risks of TB challenge trials research will be found to be justifiable. As illustrated in table 3, we contend that the estimated risks of an attenuated *M.tb* challenge trial are likely to fall below many plausible acceptable risk thresholds, as determined by the levels of voluntary risk that are deemed acceptable in other medical and non-medical contexts. Given that the estimated risks of an attenuated *M.tb* challenge trial are lower than other commonly accepted risks such as liver donation, we think the risks of the trial can be justified if their social benefits are vastly greater than the other accepted risks.

While table 3 does not include morbidity risks of comparator activities, several of the risks described have significant long-term negative health effects as well. For instance, left liver donors experience morbidity rates of 9.8%,^34^ and some kidney donors also experience post-surgery morbidities.

## Ethical considerations for a TB human challenge model

Having established that a TB human challenge model could plausibly pose acceptably low risks to volunteers while promising benefits that would exceed those risks, we turn our attention to additional and unique ethical factors pertaining to TB challenge trials. We identify five important ethical considerations of TB challenge trials that distinguish these trials from the use of challenge trials in other contexts: an exceptionally high disease burden with no highly effective vaccine, heightened third party risk following the trial, uniquely stringent biosafety requirements for the trial, risks associated with best available TB treatments and difficulties with TB disease detection. We summarise these considerations and questions for further research in table 4.

### Equitably engaging stakeholders

Experience from vaccine trials in other contexts shows that an essential requirement for an ethically conducted TB human challenge trial is active stakeholder engagement. Stakeholders include the relevant scientific community, volunteer advocacy groups, trial sponsors and community groups in TB-endemic regions. The trial team should field questions and concerns from each of these groups in advance of the trial to foster trust and collaboration throughout the study. The benefits of stakeholder engagement should be weighed against both the financial cost and social cost of delaying the potentially important results of a trial.^35^

The nature of equitable stakeholder engagement will also depend on the location of the trial. Given the fraught history of outsourcing risky medical research to low-income countries, we suggest that TB challenge trials in endemic regions should warrant exceptional scrutiny and ethical review.

### Scientific reputation

In the case of COVID-19 challenge trials, some commentators raised concerns that the ethically sensitive nature of challenge trials could harm trust in research.^36^ However, none of these concerns were grounded in context-specific empirical evidence, and the only high-powered poll that took place found broad and diverse support for these trials.^37^

While these surveys show a degree of public support for ethically sensitive challenge trials, they may not generalise to the case of TB in particular. More empirical research is needed to gauge public perception of TB human challenge trials and which trial designs receive broad support, especially for trial designs that include non-trivial risks of serious disease or death, as the occurrence of a serious incident in these trials could cause very significant damage to public trust in vaccine research.

### Fair participant selection

Like other trials, TB challenge trial recruitment will face a trade-off between trial safety (accepting young, healthy volunteers to minimise risks) and the generalisability of trial results (accepting a representative cross-section of the population).

To the extent that this trade-off exists, and it is unclear if challenge trials face a distinctive generalisability problem compared with field trials, we suggest that researchers should prioritise safety over considerations of generalisability, and thus select volunteers in the lowest risk profile, that is, young volunteers with no TB comorbidities.^38^ This is likely to reduce risks considerably, as most TB deaths occur for people that are HIV positive and/or people with low body mass indices. The elderly and very young are also at disproportionately high risk of TB disease. Notably, prescreening for healthy volunteers is limited by the possibility that a volunteer ages significantly and becomes immunocompromised after the trial, when they are still susceptible to the activation of latent TB. An attenuated model reduces this likelihood.

### Suitable site selection

Site selection for a TB human challenge trial might require unusually stringent biosafe facilities. *M.tb* is transmitted via airborne particles, and a challenge model will therefore require at least biosafety level 3 conditions.

TB may therefore face a unique tension of requiring stringent biosafety facilities which mean the trial cannot be conducted in endemic regions where the marginal risk to volunteers and third parties is lowest. If a lack of suitable facilities in endemic regions poses a problem, researchers could consider recruiting a globally diverse set of volunteers and paying for their travel to a suitable facility.

Further research is needed to weigh the various practical and ethical considerations related to optimal site selection. Other variables include certain benefits of conducting the trial in a high-income country, such as stronger medical care for participants and third parties, as well as a lower probability of siphoning already-scarce medical personnel and supplies from local communities.

### Respect for persons and autonomy

Typically, the most salient concern pertaining to autonomy and the principle of respect for persons in research ethics is how to ensure valid consent among participants. This is, of course, a salient concern for TB challenge trials, and we shall comment on this issue below. Prior to doing so though, we also wish to highlight the fact that an overly paternalistic approach to reasonable risk in research ethics may not adequately respect persons and autonomy. While the violation of autonomy would not be as serious as being compelled to undergo research or other issues that typically frighten bioethicists, we think it is substantial nonetheless.

Historically, bioethicists have sometimes dismissed the possibility that individuals are willing to take significant risks to promote the well-being of strangers. Non-directed living kidney donations, which involve both a non-trivial risk of death during surgery and an increased risk of end-stage renal disease, were prohibited until the 1960s, ostensibly for the sake of donor candidates, whose interests were over-ridden for fear that they were pathological or deranged. Now, we have good evidence to show that a considerable number of people are willing to take such risks for the public good, saving thousands of lives.

Similarly, evidence strongly suggests that individuals are willing to take significant risks in clinical trials with high social value. In a 2020 academic survey of nearly 2000 prospective volunteers for a COVID-19 human challenge trial, Rose *et al* found that the median volunteer candidate would be willing to participate in a COVID-19 human challenge trial that involved a 1% risk of death, orders of magnitude higher than the actual risk of both COVID-19 challenge studies and TB challenge studies.^39^ These volunteers were no more risk-tolerant than the general population, though they were unusually altruistic.

The number of people who would be willing to participate in a TB challenge trial should be verified empirically before investing resources into TB challenge studies. However, if sufficiently informed volunteers do come forward, we suggest that over-riding the altruism of these prospective volunteers represents a significant affront to their autonomy. Perhaps there are some harms that we should not allow individuals to autonomously expose themselves to, but we should acknowledge the costs of that claim in this context.

Of course, this argument is contingent on the assumption that such volunteers would be making autonomous decisions to participate. Developing a sufficiently robust procedure for obtaining valid consent is a significant obstacle for any research study that exposes participants to non-trivial risks. The difficulties associated with ensuring adequate understanding of information about risk and avoiding the therapeutic misconception are well-documented.^40^ These concerns are far from unique to TB challenge studies, but, when combined with the risks associated with such studies, they suggest a need for enhanced consent procedures in this context.

Notably, TB challenge may avoid one of the most widely cited obstacles to valid consent in SARS-CoV-2 challenge trials. Due to the significant uncertainties associated with the virus and the risk profiles of different demographics at early stages of the pandemic, some commentators doubted the possibility of truly informed consent to such trials.^41^ However, notwithstanding concerns about the strength of this criticism, it is far less applicable to TB challenge trials, given our better understanding of the pathogen in question.

Should these trials include the provision of financial recompense to research participants, TB challenge trials might raise the spectre of other threats to autonomy, namely coercion and undue influence. One strategy for avoiding these potential threats to autonomy is to offer only a small amount of compensation to research participants. However, even assuming that such a strategy is necessary for safe-guarding autonomy (which we doubt), it also raises salient issues of justice.

### Just payment

There are two problems with offering only a small amount of financial compensation for participation in risky research. The first is that if the amount is too low, then it may fail to cover the significant financial costs of participating in a challenge trial. To ensure equitable access to the trial, it is imperative that all those who are willing can afford to take on the financial costs that may be associated with participation.

One solution is to offer participants an amount that is necessary to cover the financial costs of their participation, although these costs are unlikely to be uniform across participants. However, this level of compensation might still constitute a form of unfair treatment in so far as such compensation would fail to recognise the various burdens that participants are taking on in the trial, not least their exposure to risk.^42^ The initial level of compensation need not include reimbursement for transportation and medical expenses, which can be reimbursed once receipts are shown.

A second concern is that even a low level of compensation may be sufficient to incentivise vulnerable individuals to participate in the trial in an exploitative manner; that is, it may take *unfair advantage* of vulnerable individuals.^43^

In short, investigators of a TB challenge trial will need to determine a level of payment that avoids financial exploitation of volunteers and ensures equitable access to research, without causing the undue inducement of vulnerable volunteers to partake in risky medical trials. Recent ethical frameworks have focused on the particular context of payment for human challenge trials. Fernandez *et al* have provided a payment worksheet for investigators to use to systematically determine an appropriate context-specific level of payment for volunteers.^44^ Given the non-trivial risk that volunteers will be incurring, there is reason to believe that undercompensation may be a greater concern than overcompensation, as some commentators pointed out in the case of COVID-19 human challenge trials.^41^

### Third party risk

Unlike COVID-19 and malaria human challenge trials, in which volunteers can be guaranteed to be non-infectious on discharge, the latent nature of TB means that trial participants may become infectious after the trial, possibly leading to third party infection. Importantly, the bulk of known risk of transmission comes from the small minority of people infected by *M.tb* who experience symptomatic disease. But there is evidence that a significant number of asymptomatic carriers may shed some level of *M.tb*. The possibility of third party risk caused by latent TB infection is one notable way in which TB human challenge models are dissimilar from recent ethical frameworks focusing on COVID-19.

**Figure 2:**
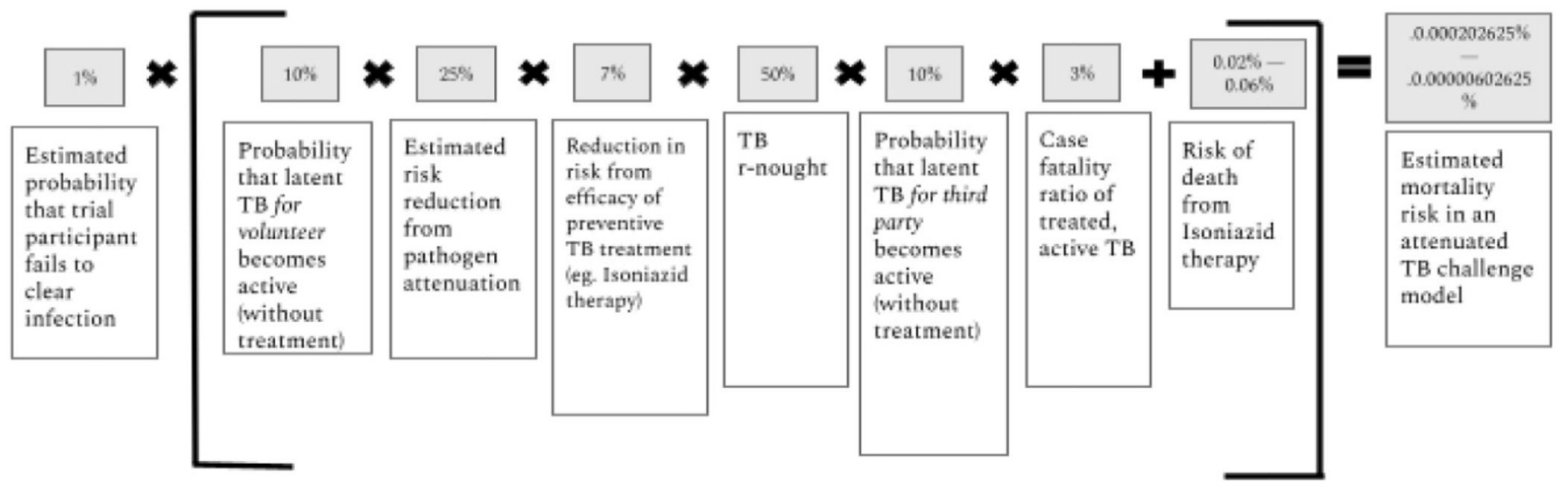
Estimated TB third party mortality from an attenuated *Mycobacterium tuberculosis* human challenge trial with 100 trial participants. TB, tuberculosis.

The likelihood of third party risk in TB challenge studies is non-zero, but it is likely to be small and controllable. Crucially, the chance that this would lead to another active TB case outside of the study is likely over an order of magnitude lower given both (1) the attenuated pathogen, (2) the low underlying probability of latent TB becoming active and (3) the availability of preventive therapy. Active monitoring of *M.tb* challenge volunteers can help ensure that they isolate if needed and follow best practices should they develop symptoms of active TB. In figure 2, we estimate the probability of third party mortality in an attenuated challenge trial with 100 participants to be 0.00000735%.

In the case of possible Zika human challenge trials, bioethicists ‘assessed the acceptability of third party risks by comparing their likelihood and magnitude to data on adverse events from similar trials that are generally viewed as ethically acceptable’, such as other human challenge trials for other diseases.^14^ As shown in table 3, TB challenge trial third party will likely fall well below other commonly accepted risks.

Despite its low probability, community TB transmission presents a unique ethical concern by potentially exposing people to risk who are not enrolled in the study as research subjects. Ethical principles for managing risks to populations not involved in the trial itself can be borrowed and adapted from other studies that pose diffuse risks to communities, such as field trials of genetically modified disease-resistant mosquitoes.

These principles include only running a trial when the targeted disease is a public health problem in the area in which it is conducted, the benefits to the community are likely to outweigh the risk, community leaders approve of the trial and measures are put in place to protect the health of at-risk community members.^45^ We note, however, that the principle of running a TB challenge trial in an endemic region may be difficult in the short-term due to a lack of ample Biological Safety Level-3 challenge facilities in endemic regions. Additionally, more modelling is needed here, as it is possible that worse socioeconomic conditions in endemic regions may increase transmission to such an extent that will outweigh the lower marginal risk of disease in these regions.

On a theoretical level, we at the very least endorse Nir Eyal’s ‘low-hanging fruit’ approach, outlined in the context of HIV trials with antiretroviral therapy interruption, which says that in light of bioethicists’ significant uncertainty of the moral status of third parties in comparison to trial participants, ‘if a measure affords substantial protections to nonparticipants, it costs little time, effort, and money, and it involves no independent transgressions (eg, of participants’ privacy), then we can tentatively conclude that this measure is mandated for both ethical and regulatory purposes’.^46^

We invite further research into these questions. Researchers should model as much as possible the exact risks to third parties based on different trial designs, as well as possible mechanisms for treatment should any third parties fall ill. On the latter, ethicists should try to determine the extent to which those running the trial ought to provide different types of care to third parties.

## Virulent TB human challenge trials

Using attenuated *M.tb* as a challenge agent would reduce risks to volunteers and third parties considerably in a challenge trial compared with virulent *M.tb*; however, it may do so at the cost of reducing the biological relevance, and in turn, the public health value, of the challenge model. In particular, the less a challenge agent mimics a virulent pathogen, the lower the probability that the challenge agent can be used to speed vaccine authorisation or help discern immune correlates of protection for the virulent pathogen. If challenge trials with attenuated *M.tb* would be uninformative for vaccine research, then putting volunteers at risk in a trial would be unethical and a poor use of scientific resources.

In such circumstances, could there be a case for a challenge trial using a virulent challenge agent, given the higher risks involved? Prima facie, it might be argued that there could be some considerable benefits to such a design. For instance, a virulent challenge agent may provide more decisive evidence of early-stage vaccine efficacy, and therefore may put promising vaccine candidates on a more expedited path to field trials than an attenuated challenge agent. A virulent model could also plausibly be more easily combined with other data to provide a sufficient basis for vaccine licensure. Lastly, a virulent model could in principle be used to validate suspected correlates of protection observed in attenuated models.

However, this prima facie view does not provide a straight-forward justification of such a design. First, these benefits should not be overstated. An attenuated *M.tb* challenge model may be close enough to virulent *M.tb* to capture many of the benefits. We invite further research from commentators on the degree to which attenuated *M.tb* could mimic virulent *M.tb* by comparing different methods of attenuation. Additionally, as we explained in a previous section, a well-established attenuated *M.tb* challenge model could also be used to bridge human and animal vaccine studies in virulent animal models. Accordingly, a trial using a virulent strain may not be necessary.

Most importantly though, the prima facie case is significantly weakened by the greater costs of conducting a virulent model: trial volunteers would be put at greater risk of post-trial activation of latent TB infection. If trial participants or community members are immunocompromised, this risk, while not wholly dissimilar to other risks taken in public health contexts (see table 3), would be considerable.

If attenuated models can capture significant public health value, then virulent trials would be unnecessary and unethical. If attenuated models face significant limitations—for instance, by proving to be technically infeasible or significantly dissimilar from the virulent pathogen such that they are ineffectual for vaccine testing—then trials with the virulent pathogen might yet warrant consideration. However, the case in their favour is likely to be far less convincing due to the higher risks that such a trial would involve.

## Conclusion

Human challenge trials are a critical tool in the toolbox in fighting infectious diseases that take millions of lives annually, and they may be particularly valuable in pandemic scenarios that render rapid field trials implausible. Especially as advances in messenger RNA vaccine technology shorten the timeline for vaccine design and manufacture, clinical trials will pose the primary bottleneck for getting life-saving vaccines to market, making the role of challenge trials even more important.

In the case of TB, challenge trials may be an essential step in ridding the world of a disease that has caused untold suffering since the time of Pharaohs. Ethicists should continue to develop frameworks on acceptable levels of risk for human challenge trials to reduce uncertainty and friction in the decision-making process. To the extent possible, these estimates should be quantified and made commensurable by being expressed in similar terms, such as expected lives saved/lost or expected disability-adjusted-life-years saved/lost.

We contend that in further research, due attention must be paid to the growing evidence that fully informed and consenting individuals are willing to take on altruistic risk in medical contexts to advance research

More rigorous modelling must be done to discern the risks and benefits of different TB challenge models, and to answer important ethical questions surrounding third party risk and just payment. In this article, we have begun an analysis of this important topic, and believe that if these trials would significantly accelerate TB vaccine development, there is good reason to consider conducting challenge trials with attenuated strains like BCG or attenuated *M.tb*.

## Supplementary Material

This content has been supplied by the author(s). It has not been vetted by BMJ Publishing Group Limited (BMJ) and may not have been peer-reviewed. Any opinions or recommendations discussed are solely those of the author(s) and are not endorsed by BMJ. BMJ disclaims all liability and responsibility arising from any reliance placed on the content. Where the content includes any translated material, BMJ does not warrant the accuracy and reliability of the translations (including but not limited to local regulations, clinical guidelines, terminology, drug names and drug dosages), and is not responsible for any error and/or omissions arising from translation and adaptation or otherwise.

Supplementary Material

## Figures and Tables

**Table 1:** Estimated lives saved by a TB challenge model over the next 10 years*

	Probability that a new TB challenge model speeds new TB vaccine authorisation relative to other trial designs by reducing uncertainty in early stage vaccine development†	Years that a new TB challenge model saves in authorising new TB vaccine relative to other trial designs	Risk difference in mortality between new authorised TB vaccine and current standard of vaccination	Percentage of population in endemic regions who receive vaccine^47^	Estimated lives saved over next 10 years by using new TB challenge model‡
Low-end estimate	0.1	3	0.1	0.8	26 400
Middle-end estimate	0.25	4	0.3	0.8	264 000
High-end estimate§	0.5	5	0.5	0.8	1 100000

The footnote symbols used in the table to justify assumptions can be referred to online supplemental file 1. TB, tuberculosis.

**Table 2:** Use cases, limitations and risks of different types of TB challenge trials

TB challenge model	Advantages of the model	Scientific limitations	Risks to volunteers
Intradermal BCG challenge model	▶ Proven safety and feasibility at a low and higher dose by Minhinnick *et al.*^29^ ▶ Can be used as a similar proxy for *M.tb* since BCG has >99% sequence to *M.tb* at nucleotide level. ▶ To the extent that BCG mimics *M.tb,* can be used to identify biosignatures of TB risk, explore TB pathogenesis and test in-the-pipeline TB vaccines.	▶ BCG is not generally a pathogenic strain that causes typical TB and lacks critical virulence genes. ▶ Target antigens unique to *M.tb* would not be suitable for the BCG challenge model. ▶ Intradermal model administration may fail to mimic pulmonary delivery.	▶ Extremely low risks of short-term mortality and disease, long-term sequelae and community transmission, as demonstrated by Minhinnick *et al.*^29^
Aerosol BCG challenge model^10 48^	▶ Proven safety and feasibility by Davids *et al.*^49^ ▶ Can be used as a similar proxy for *M.tb* since BCG has >99% sequence to *M.tb* at nucleotide level. ▶ To the extent that BCG mimics *M.tb,* can be used to identify biosignatures of TB risk, explore TB pathogenesis and test in-the-pipeline TB vaccines.	▶ BCG is not generally a pathogenic strain that causes typical TB and lacks critical virulence genes. ▶ Target antigens unique to *M.tb* would not be suitable for the BCG challenge model. ▶ Confirmation that BCG challenge reflects pulmonary vaccine effect may ultimately require pulmonary challenge trials and comparison of validated immune correlates of protection.^50^	▶ Extremely low risks of short-term mortality and disease, long-term sequelae and community transmission, as demonstrated by Davids *et al.*^49^
Intradermal *M.tb* challenge model^6^	▶ Greater biological relevance than BCG. ▶ Detection of TB is more feasible than pulmonary *M.tb* challenge models.	▶ Less biologically relevant than pulmonary model, and therefore unclear if sufficient to determine immune correlates of protection and downselect vaccine candidates.	▶ Significantly lower safety concerns than attenuated and virulent pulmonary models.
Attenuated pulmonary *M.tb* challenge model^6^	▶ Depending on level and type of attenuation, may balance safety concerns and biological relevance.	▶ Feasibility issues, including a lack of an attenuated strain and difficulty detecting the bacterial burden.	▶ Significantly lower than a virulent *M.tb* model, depending on level of attenuation.
Virulent *M.tb* challenge model^6^	▶ Most biologically relevant challenge model.	▶ Feasibility issues, most notably difficulty detecting bacterial burden.	▶ Significant long-term sequelae associated with cured TB and TB treatments. ▶ Slight risk of community transmission from late reactivation due to failure to eradicate TB.

BCG, Bacillus Calmette-Guérin ; *M.tb, Mycobacterium tuberculosis* ; TB, tuberculosis.

**Table 3:** TB challenge trial compared with other common procedures and risks

Activity	Micromorts (one in a million risk of death)
*Total third party risk in an attenuated M.tb human challenge* *trial* (figure 2)	*2–6*
*Attenuated M.tb human challenge trial* (figure 1)	*2–66*
Driving to New York from Los Angeles and back^51^	22
Living kidney donation^52^	310
’Brazilian butt lift’ cosmetic surgery^53^	435
Motorcycling to New York from Los Angeles and back^51^	897
Right liver lobe donation^34^	4000

M.tb, Mycobacterium tuberculosis .

**Table 4:** Ethical considerations for TB human challenge trials compared with recent human challenge trials

	Tuberculosis	COVID-19	Zika	Malaria	Hepatitis C	Unique ethical considerations for a TB human challenge model:
Risks to volunteers	▶ Attenuated trial: between 2.6–6.6 in 1 000 000 (table 3).	▶ 8.2 in 100 000 risk of death in trial.^54^	▶ Risk of Guillain- Barré syndrome as high as 2/10 000.^55^ ▶ Later exposure to related viruses could compound risks.	▶ Influenza-like symptoms may appear, but laboratory strain is very susceptible to drugs. No deaths or severe adverse reactions in years of malaria trials.	▶ Risk of hepatitis C symptoms, such as fever and fatigue. Possible risk of coinfection with other blood-borne pathogens.	▶ Risks to volunteers in a TB challenge model may be higher than recent human challenge trials. How can these risks be minimised, and is the risk-benefit ratio favourable?
Disease burden and availability of vaccines	▶ ∼1.5m deaths per year. ▶ No very effective vaccine.	▶ ∼3 m deaths per year. ▶ Several highly effective vaccines exist.	▶ ∼51 total deaths between 2016 and 2019. ▶ Effective vaccine recently licensed by WHO.	▶ ∼400 k deaths per year. ▶ No very effective vaccine.	▶ ∼290 k deaths per year. ▶ No very effective vaccine.	▶ TB may uniquely have an extremely high disease and no very effective vaccine. How should bioethical analysis change in light of the uniquely high social value of accelerating TB vaccine development?
Site selection	▶ High level of biosafety needed due to airborne pathogen. ▶ Benefits of endemic vs non-endemic region must be weighed.	▶ Biological Safety Level-3 laboratories at Imperial College London and Oxford University.	▶ Biosafety needed, though Zika is not airborne, so plausibly BSL 2 is sufficient. ▶ Researchers leaned toward conducting in a non-endemic region.^55^	▶ Biosafety needed, though malaria is not airborne, so plausibly BSL 2 is sufficient. Conducted in both endemic regions and non-endemic regions.^56^	▶ Biosafety needed, though hepatitis is not airborne, so plausibly BSL 2 is sufficient. Not yet decided, though would likely be in Canada given the major hepatitis research hubs located there.	▶ TB may have the unique combination of being airborne and endemic to certain regions. Biosafe laboratories do not exist in ample supply in endemic regions, where marginal risk to volunteers is minimised. How should ethicists deal with this tension?
Disease detection	▶ Pulmonary challenge model is difficult to detect, intradermal model easy to detect.	▶ Easy disease detection through lateral flow assay and PCR.	▶ Easy to detect, though often conflated due to cross-reactivity with flaviviruses. ▶ Detection is done through PCR.	▶ Easy to detect via thick smear microscopy showing parasites in blood.	▶ Easy to detect via PCR blood test to find active hepatitis.	▶ Pulmonary TB may be uniquely difficult to detect compared with other diseases studied with human challenge trials. What implications does this have for the design and social value of a TB challenge model?
Third party risk	▶ Plausibly significant: Latent TB may cause community transmission post-trial.	▶ Near zero: Volunteers are quarantined and must test negative before release.	▶ Plausibly significant: Possibility of transmission to childbearing-capable sexual partners and their fetuses.	▶ Near zero: Volunteers are quarantined and must test negative before release.	▶ Plausibly significant: Possibility of transmission to sexual partners during viraemic period, 4–6 months.	▶ Trial volunteers developing latent TB may give a unique need for trialists to ensure community protections for volunteer communities. How can third party risk be minimised through trial follow-up?
Availability of treatments	▶ Effective treatments exist, such as isoniazid, though with non-trivial risk of hepatotoxicity.	▶ Safe, effective treatments exist, such as remdesivir.	▶ None.	▶ Save, effective treatments exist. Chloroquine plus other safe standard drugs, depending on trial, eg, artemether, lumefantrine and primaquine.	▶ Save, effective treatments exist. Either sofosbuvir-velpatasvir or glecaprevir- pibrentasvir cocktail, with optional retreatment.	▶ The treatments for TB may involve greater risks than other diseases recently studied in human challenge trials. How can these treatment risks be minimised?

TB, tuberculosis.

## Data Availability

No data are available.
