# Supplementary material for: Exploring the ethics of tuberculosis human challenge models
Source: J Med Ethics. 2023 Dec 30;52(4):e109234. doi: 10.1136/jme-2023-109234 (PMC7617737; doi:10.1136/jme-2023-109234)
Supplement: online supplemental file 1 [file jme-52-4-s001.pdf]

APPENDIX A: TABLE 1 ASSUMPTIONS

* In the absence of rigorous modeling of TB deaths through 2030 following the COVID-19 pandemic, we optimistically (and therefore, in the context of our model, conservatively) assume that TB deaths decrease by 4% per year over the next ten years from the estimated 1.4 million deaths in 2019. We also assume 80% coverage of a new licensed TB vaccine in endemic regions based on current coverage rates of the BCG vaccine in Africa [56]

† The reasoning for why challenge trials can increase the probability of trial success is explained in the section *The state of TB vaccine development and TB human challenge trials.* The justification for the .1 to .5 range in particular is corroborated in Figure 1 of Pollard et. al 2020.

‡ Calculated as follows: (Probability that a new TB challenge model speeds new TB vaccine authorization relative to other trial designs) x (Years that a new TB challenge model saves in authorizing new TB vaccine relative to other trial designs) x (Difference in reduction of mortality between new authorized TB vaccine and next best alternative) x (Expected average lives lost per year globally from TB)

§ Only 49% of infectious disease vaccines in Phase 2 trials ultimately receive FDA authorization (Wong et al. 2019). The average timeline for a new vaccine is 10.71 years, with an overall market entry probability of 6% (Sekhar and Kang 2020). The average costs are between $200 million and $900 million (Roestenberg et al. 2018). The typical Phase 3 trial lasts over 5 years for TB 2-4 years, unlike a challenge trial, which typically lasts 1-3 months (Sekhar and Kang 2020). Phase 1 and 2 testing typically lasts 2-3 years at a minimum. Given limited resources for Phase 3 trials for TB vaccines, a TB challenge trial could shorten the path to vaccine authorization by the typical duration of a Phase 3 trial in one of three ways (Roestenberg et al. 2018):

a) Promising results from a TB human challenge trial could secure funding for a Phase 3 trial that otherwise would not have received funding. The urgency of additional funding for TB vaccine candidates is described in the section *The state of TB vaccine development and TB human challenge trials.*
b) Results from a multi-arm challenge trial could advance the most promising TB vaccine to Phase 3 trials, which otherwise would have been deprioritized given limited resources. This method, which has been successfully used in the cases of malaria and dengue, is described in the section *The state of TB vaccine development and TB human challenge trials*. In particular, challenge trials can help with the identification of immune correlates of protection which can then be validated in field efficacy trials and subsequently used for licensure. They can also help with dose optimisation and regime selection.
c) In exceptional cases, efficacy data from a TB human challenge trial combined with data from a safety trial may be sufficient on its own for vaccine licensure. This occurred in the case of Vaxchora, which has a similar global burden to TB.
